# Rabies outbreak in Lefeisa town, Somali region, Ethiopia: A report on epidemiological trends and one health implications

**DOI:** 10.1016/j.onehlt.2025.101165

**Published:** 2025-08-19

**Authors:** Hassan Abdi Hussein, Abdilahi Adan Ahad, Mohamed Hassen Abdilahi

**Affiliations:** a _College of Veterinary Medicine, Department of One Health in Tropical Infectious Disease, Jigjiga University, P.O. Box: 1020, Jigjiga, Ethiopia_; b _Health Bureau, Somali Regional State, P.O. Box 228, Jigjiga, Ethiopia_; c _Pastoral Development Bureau, Somali Regional State, P.O. Box 795, Jigjiga, Ethiopia_

**Keywords:** Rabies outbreak, Dogs and Wild animals, One health, Zoonotic diseases, Epidemiology, Public Health, Lefeisa, Somali, Ethiopia

## Abstract

***Background***: Rabies is a significant zoonotic disease that poses a severe public health threat, particularly in pastoralist communities where human-animal interactions are frequent. Understanding outbreak dynamics is critical for developing effective control strategies and advancing sustainable health interventions.

***Objectives***: This study aimed to describe the epidemiological characteristics of a rabies outbreak that affected both human and animals in Lefeisa town and to highlight the implications for a coordinated One Health approach to disease control and prevention.

***Methods***:Outbreak data were collected from health facilities and official reports in Lefeisa town for the period of May to September 2023. The analysis included human and animal cases suspected of rabies. Diagnosis in animals was confirmed using a rapid diagnostic kit. Descriptive statistics were used to summarize the epidemiological trends of the outbreak.

***Result***: A total of 28 people and 19 animals suspected of rabies were documented, of which two animals were confirmed using rapid diagnostic kit, with dogs identified as the main reservoir of the virus. Among the suspected human cases, 57.1 % occurred in individuals aged <15 years, and 71.4 % were male. Most bite exposures were classified as Category-3 (82.1 %). While the majority of bitten individuals have survived 85.7 %, a case fatality rate of 7.1 % was recorded and the outcome of an additional 7.1 % remains unknown. Confirmed cases of rabies were found in dogs and donkeys, while other species were classified as suspected based on clinical signs. Notably, community members often resorted to killing suspected rabid animals due to fear and sense of urgency to prevent potential harm, even before formal investigations could take place.

***Conclusion***: These findings underscore the crucial need for integrated One Health strategy that combines improved mass dog vaccination, community education on bite prevention and management, and enhanced diagnostic and surveillance capabilities to prevent future outbreaks and protect both human and animal health.

## Highlights

• Rabies outbreak in Lefeisa Town, Somali Region (May–Sep 2023) led to 28 human exposures and 19 affected animals in Ethiopia.
• Children <15 and males were the most affected; majority of bites were high-risk Category III.
• Dogs served as the primary reservoir, with laboratory-confirmed rabies cases in both dogs and donkeys.
• Outbreak peaked in July 2023; locals killed suspected rabid animals before investigation, hindering surveillance.
• Findings highlight urgent need for dog vaccination, public education, and stronger One Health surveillance systems.

## 1 Introduction

Rabies is a fatal zoonotic viral disease caused by the rabies virus, which belongs to the genus *Lyssavirus* and the family *Rhabdoviridae*. The virus is a multi-host pathogen capable of infecting all warm-blooded animals [1]. Dogs and wild carnivores such as foxes, jackals, raccoons, skunks, and bats are the main carriers of the rabies virus. In endemic regions, rabid dogs are the primary source of human infections, underscoring their critical role in transmission [2].

The global burden of rabies remains substantial, causing an estimated 59,000 deaths annually [3]. While a reduction of death in human due to rabies has been actualized in many locations from controlling the disease in canine populations [4], deaths still occur in numerous countries due to lack of robust disease surveillance, limited access to appropriate post-exposure prophylaxis (PEP), and under-appreciation of the public health implications of the disease [5]. Populations in low setting countries are disproportionately affected triggering significant economic hardship through PEP costs, lost productivity, and livestock losses [6].

In Ethiopia, foxes, skunks, and spotted hyenas are known reservoirs of rabies, with the disease also reported in Ethiopian wolves, yellow mongooses, and leopards [7]. However, rabies is primarily transmitted to people through the bites of infected dogs, and the country has a high incidence of rabies-related deaths, ranking second in Africa, prompting experts to recognize it as the country's top priority zoonotic disease [8]. The absence of robust surveillance systems has led to significant inaccurate estimates of disease burden. However, existing studies indicate that about 97,000 people need post-exposure prophylaxis each year, costing approximately USD 2 million. Moreover, the estimated number of fatalities from rabies is approximately 3000 annually [9].

A total of 37,989 suspected human rabies exposures and 297 deaths were reported in 2018–2022, averaging 7598 cases and 59 deaths annually, with incidence rates of 6 and 0.05 per 100,000 population, respectively [10]. A recent meta-analysis estimated the pooled prevalence of rabies in Ethiopia at 32 %, with individual study findings ranging from 1 % to 78 %. Subgroup analyses indicated prevalence rates of 28 % in animals and 33 % in humans. Regional disparities were notable: prevalence was highest in Addis Ababa (78 %) and lowest in the Amhara Regional State (5 %). No eligible data were available from the eastern (Somali region in particular) and southern regions of the country [11]. Annual rabid dog exposures in selected urban and rural districts were estimated at 135 and 101 bites per 100,000 inhabitants, respectively [12].

The most effective strategy to control rabies in Ethiopia, and globally, is through mass dog vaccination campaigns combined with a multi-sectoral approach. This approach focuses on preventing human exposure by addressing the disease at its source—primarily through controlling rabies in animal populations, particularly dogs, which are the main reservoir and transmitter of the virus. By integrating efforts across human, animal, and environmental health sectors, this strategy ensures a comprehensive and sustainable response to rabies prevention and control [13].

The One Health (OH) approach is crucial for combating rabies in Ethiopia by highlighting the interconnectedness of human, animal, and environmental health. Key strategies include implementing mass dog vaccination campaigns, integrated bite case management (IBCM) training to frontline health workers, increasing public education on bite prevention, and improving access to PEP [14]. The National One Health Strategy Plan encourages cooperation among veterinarians, healthcare professionals, and environmental experts by focusing on mass dog vaccinations and raising public awareness initiatives. By coordinating these efforts, Ethiopia aims to reduce rabies transmission, protect vulnerable populations, and work towards eliminating human rabies deaths by 2030 [15].

Due to the absence of routine laboratory surveillance and limited vaccination of dogs in remote and rural areas, rabies outbreaks in Ethiopia leads to significant human and animal losses, and a considerable economic impact. In May 2023, a rabies outbreak occurred in Lefeisa Town, a small town located on the eastern part of Ethiopia, in Somali regional state, resulting in numerous cases among both humans and animals. This study aims to describe the epidemiological characteristics of the outbreak, evaluate the response, and offer actionable recommendations for future prevention efforts.

## 2 Methodology

### 2.1 Study setting

**Fig. 1:**
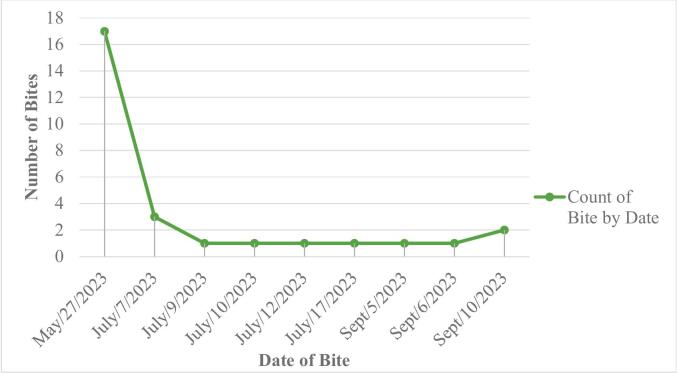
Overview of the timeline of suspected rabid animal (dog) bite cases.

Lefeisa (Somali: Lafaciise) is a town in eastern Ethiopia, located within the Fafan Zone of the Somali Region. It is self-administering city council but was previously part of Awbare district and is situated approximately 32 km north of Jigjiga and 664 km east of Addis Ababa (see Fig. 1). Geographically, Lefeisa is positioned at 9° 36′ 34″ N latitude and 42° 58′ 50″ E longitude, at an elevation of 1609 m above sea level. The estimated population of the town and its surrounding areas is approximately 120,000. Lefeisa experiences a semi-arid climate characterized by temperatures ranging from 15 °C to 30 °C and receives an average annual rainfall of 880 mm.

Residents of Lefeisa primarily engage in a sedentary agrarian lifestyle, cultivating crops like wheat, sorghum, maize, and various vegetables suited to the local climate. Livestock rearing, particularly of cattle, sheep, and goats, is also vital to the community's economy. However, these agricultural and herding activities are closely tied to seasonal rainfall, making the population susceptible to climate variability and drought. Additionally, Lefeisa has limited healthcare infrastructure, with only single health center available and a minimally functional veterinary clinic for livestock treatment. A significant challenge to disease control, particularly rabies, is the presence of a large population of stray dogs that roam freely throughout the town, serving as a potential primary source for rabies. In contrast, only a small number of dogs are owned by farmers, primarily for livestock guarding in the town's periphery. This disparity in dog ownership and management, combined with climatic challenges and inadequate resources, heightens the community's vulnerability and complicates the control of zoonotic diseases.

### 2.2 Study design

A cross-sectional study was conducted to collect snapshot data on individuals affected by the outbreak and the animals involved.

### 2.3 Data collection

#### 2.3.1 Case definition

To promote standardized reporting and ensure consistent classification of cases during the outbreak investigation, the following definitions were used, based on WHO criteria and adapted for local context [25].

##### 2.3.1.1 Suspected human rabies case

Any individual who has been bitten or scratched by an animal—particularly a dog or wild carnivore—showing symptoms suggestive of rabies, such as fever, agitation, unprovoked aggression, hypersalivation, or neurological signs (e.g., hydrophobia, paralysis), should be classified as a suspected rabies case and evaluated promptly.

##### 2.3.1.2 Probable human rabies case

A suspected case with documented exposure to a suspected or confirmed rabid animal and presenting clinical signs consistent with rabies, but without laboratory confirmation and no alternative diagnosis.

##### 2.3.1.3 Confirmed human rabies case

A suspected or probable case with laboratory confirmation of rabies virus infection using diagnostic methods such as RT-PCR, direct fluorescent antibody (DFA) test, or viral isolation from saliva, cerebrospinal fluid (CSF), or brain tissue.

##### 2.3.1.4 Suspected animal rabies case

Any animal—especially dogs—that shows sudden behavioral changes, unexplained aggression, hypersalivation, unprovoked attacks, or neurological signs, and dies within 10 days or is euthanized.

##### 2.3.1.5 Confirmed animal rabies case

An animal with laboratory confirmation of rabies virus infection through DFA or RT-PCR testing on post-mortem brain tissue. All cases were documented using a standardized rabies reporting form and linked to epidemiological data for further analysis.

#### 2.3.2 Outbreak epidemiological data collection

Epidemiological data on rabies related cases were collected from records at the Lefeisa Health Center. Information on patient demographics (age and sex), the date of the bite, the species of the biting animal (e.g., dog, cat, wild animal), rabies vaccination history, and the clinical outcome (survival or death) were retrieved.

### 2.4 Consent statement

Verbal informed consent was obtained from all participants or their guardians prior to data collection. Given the urgency of the outbreak and the need for rapid data collection, verbal consent was deemed appropriate and was documented by the data collectors. Community leaders were informed about the study objectives and provided their support. Efforts were made to ensure the privacy and confidentiality of all participants throughout the research process. All data collected has been handled with the utmost care to protect the identities and personal information of those involved.

### 2.5 Ethical approval

Ethical approval was obtained from the Research Ethics Committee of Jigjiga University, which reviewed the proposal in accordance with established rules and regulations and granted approval.

### 2.6 Data analysis

The collected data were analyzed using descriptive statistics. Frequencies and percentages were calculated to summarize the distribution of rabies cases by patient demographics (age, sex, residence), biting animal species, and case outcome. The date of bite information was used to describe the temporal pattern of the outbreak. Data were organized and presented in tables and figures.

## 3 Results

### 3.1 Suspected rabies exposure in human cases

On May 27, 2023, a suspected rabid dog initiated a series of bite incidents in Lefeisa town. The first incident involved an eight-year-old girl, who was bitten on the hand. A 29-year-old man who attempted to assist the girl was also bitten on the leg. Throughout that day, the dog continued to bite individuals, resulting in 17 residents of Lefeisa town being bitten by late the evening. The dog, exhibiting uncontrolled behavior, was subsequently killed. The origin of the dog remains unknown, and their carcass was buried before medical teams could obtain samples. All of the 17 individuals who sustained Category-3 bites were transported to the Lefeisa Health Center. Following the initial wound cleaning, they were referred to Jigjiga University Sheikh Hassan Yabare Hospital, located 32 km to the south, for post-exposure prophylaxis (PEP) administration and further treatment. Under ideal conditions, the transfer to the hospital typically takes around two hours. However, rough road conditions and occasional traffic congestion can cause significant delays. When including factors like ambulance wait times or unavailability due to other emergencies, the transportation duration can extend considerably—sometimes up to 24 h. These logistical challenges underscore the urgent need for enhanced emergency response infrastructure in the area.

Among the bite victims were a three-year-old girl and a 60-year-old woman, both of whom sustained severe bites to the neck. Tragically, both individuals presented clinical signs consistent with rabies encephalitis, including dysphagia and hydrophobia, progressing to coma and ultimately resulting in death on June 11 and June 19, 2023 respectively.

Further bite incidents occurred in Lefeisa town in July and September 2023. On July 7, 2023, a suspected rabid dog bit three individuals. Four separate dogs bit four people on July 9, July 10, July 12, and July 17, 2023. In September, four cats were involved in bite incidents on September 5, September 6, and September 10, 2023 (see Fig. 1). (See Table 1, Table 2.)

**Table 1:** Descriptive Summary of animal (dog) bite cases in Lefeisa.

Variable	Category	Frequency	Percent (%)
Age	<15 Years	16	57.1
	16–59 Years	10	35.8
	>60 Years	2	7.1
Sex	Male	20	71.4
	Female	8	28.6
Biting Animal	Dog	24	85.7
	Cat	4	14.3
Category of Bite	Category-1	4	14.3
	Category-2	1	3.6
	Category-3	23	82.1
Site of Bite	Leg/Thigh	17	60.7
	Hand/Shoulder	8	28.6
	Head/Neck	3	10.7
Admission Status	Admitted	22	78.6
	Not Admitted	6	21.4
Outcome	Recovered/Survived	24	85.7
	Death	2	7.1
	Unknown	2	7.1

**Table 2:** Overview of animals involved in suspected rabies outbreak in Lefeisa.

Animal Involved	No. Suspected	No. Killed	No. Died	No. Confirmed
Dog	6	6		1
Cat	4	-⁎	–	–
Cow	2		2	
Goat	1		1	
Donkey	2	1	1	1
Hyena	2	2		
Fox	1	1		
Total	18	10	4	2

Animals classified as ‘Confirmed’ have received a positive diagnosis through testing, while those labeled as ‘Suspected’ did not undergo testing, leaving their status undetermined.

⁎ Cats outcome was unknown, so not counted in “Died”. No. stands for Number.

The majority of cases affected younger individuals; those aged <15 years represented 57.1 % of cases. A disproportionate number of cases occurred in males, comprising 71.4 % of cases. Dog bites were the primary source of exposure, accounting for 85.7 % of incidents. The majority of bites were classified as high-risk (Category-3), indicating a substantial risk of rabies transmission. The most common bite location was the leg/thigh (60.7 %). The majority of patients sought hospital care, with 78.6 % requiring admission. While the majority of cases resulted in survival (85.7 %), there were two fatalities (7.1 %) and two unknown status (7.1 %).

### 3.2 Rabies outbreak in animal and initial response

The suspected rabies outbreak in Lefeisa, Ethiopia, presented a stark picture of community response in the face of a deadly zoonotic disease. Between May and September 2023, a series of animal cases were documented, revealing the extent of the suspected infection. Predominantly, dogs were identified as the primary source of concern, with six dogs suspected of rabies being killed by community members between May 27th and July 12th due to aggressive and erratic behavior that included biting incidents involving both people and other animals. On July 17th, one dog was confirmed to have rabies by joint outbreak investigating team from Jigjiga Regional veterinary Laboratory using a BioNote rapid antigen detection test and was subsequently euthanized. This confirmation validated the community's fears and actions, although it also raised ethical considerations regarding the preemptive killing of animals without confirmed diagnoses.

The outbreak was not limited to dogs. Donkeys were also affected, with one suspected case dying on July 8th and another confirmed case being euthanized on July 19th, both after being bitten by suspected rabid dogs. Similarly, two cows succumbed to the disease on July 20th, having also been bitten by suspected dogs. A goat suspected of rabies had an unknown outcome as of July 22nd, highlighting gaps in follow-up and reporting. Wild animals were also implicated, with two hyenas killed on June 23rd and 24th, and a fox on July 25th, after they were observed behaving unusually during daylight hours, a sign of potential neurological disturbance. In September, four cats were suspected of rabies following minor scratches inflicted on children, though their outcomes remained unknown.

The data reveals a clear pattern: most animals were classified as rabies cases based on observed behavioral changes or exposure to rabid animals, with only a few cases confirmed through testing. The majority of these suspected animals were either killed by the community or died naturally, while some cases, particularly involving goats and cats, lacked follow-up, leaving their outcomes uncertain. The timeline of the outbreak shows a peak in cases during July 2023, coinciding with the height of the outbreak. This summary underscores the widespread impact of rabies across multiple species and the challenges of managing such outbreaks in resource-limited settings. It also emphasizes the urgent need for improved surveillance, humane animal handling practices, and stronger public health interventions to effectively address rabies and prevent future outbreaks in the community.

## 4 Discussion

The findings from the rabies outbreak in Lefeisa reveal critical insights into the distribution and demographic patterns of the disease, which are essential for understanding its epidemiology and guiding targeted interventions. The age distribution of cases underscores the vulnerability of younger individuals to rabies. A significant majority of cases (57.1 %) occurred in the 0–15 years age group, indicating that children are disproportionately affected. This trend aligns with global observations that children are at higher risk due to their frequent interactions with animals and limited awareness of rabies prevention measures [16]. The 18–59 years age group accounted for 35.8 % of cases, while individuals older than 60 years represented only 7.1 % of cases. These findings emphasize the importance of implementing educational programs in schools and communities to raise awareness about rabies prevention, particularly among children and their caregivers [17].

The sex distribution of cases reveals a notable disparity, with males accounting for 71.4 % of cases compared to 28.6 % in females. This gender imbalance may be attributed to differences in exposure risk, as males are often more involved in outdoor activities, such as farming or herding, which increase their likelihood of encountering rabid animals. Additionally, cultural or behavioral factors may contribute to this disparity. Public health interventions should consider these gender-specific risks and tailor awareness campaigns to address the higher exposure among males [18].

Dog bites were the primary source of rabies exposure, accounting for 85.7 % of cases (24 cases), while cat bites represented 14.3 % of cases (4 cases). This finding aligns with global data indicating that dogs are the main reservoir and transmitter of rabies, particularly in endemic regions [4,9]. The predominance of dog bites highlights the importance of implementing robust dog vaccination programs and population control measures. Vaccinating at least 70 % of the dog population has been shown to effectively interrupt rabies transmission, as demonstrated in successful control programs in countries like Sri Lanka and Tanzania [14,19]. Additionally, public education campaigns on responsible pet ownership and avoiding stray animals are essential to reduce human exposure [20].

The majority of bite wound were classified as Category-3 (high risk), accounting for 82.1 % of cases (23 cases). Category-1 (low risk) and Category-2 (moderate risk) bites represented 14.3 % (4 cases) and 3.6 % (1 case), respectively. The high proportion of Category-3 bites, which involve deep wounds or mucous membrane exposure, underscores the significant risk of rabies transmission in these cases [21]. This finding emphasizes the need for immediate PEP, including wound cleaning, rabies vaccination, and administration of rabies immunoglobulin (RIG), to prevent the onset of clinical rabies. Strengthening healthcare systems to ensure the availability and accessibility of PEP in rural and underserved areas is critical [22]. The most frequent site of bites was the leg/thigh, accounting for 60.7 % of cases (17 cases), followed by the hand/shoulder (28.6 %, 8 cases) and the head/neck (10.7 %, 3 cases). The location of bites is a key factor influencing the speed of rabies virus transmission to the central nervous system. Bites to the head and neck are particularly dangerous due to the proximity to the brain, which can lead to faster disease progression. However, the predominance of leg/thigh bites in this outbreak may reflect the typical interactions between humans and animals, such as dogs biting individuals while they are walking or working outdoors. Public health messaging should emphasize the importance of seeking immediate medical care for all animal bites, regardless of location, to mitigate the risk of rabies [21].

The high rate of hospital admissions (78.6 %, 22 cases) suggests that most individuals in this area sought formal medical care, in contrast to regions where a lower perceived risk of animal bites and a reliance on traditional healers prevails [23]. However, 21.4 % of cases (6 cases) were not admitted, which raises concerns about barriers to healthcare access, such as cost, distance, or lack of awareness. Addressing these barriers is essential to ensure that all bite victims receive timely and appropriate care [24]. In terms of outcomes, 85.7 % of cases (24 cases) recovered or survived, while 7.1 % (2 cases) resulted in death, and the outcome was unknown for 7.1 % (2 cases). The occurrence of deaths, though relatively low, underscores the lethality of rabies once clinical symptoms develop. These fatalities highlight the importance of early intervention, including prompt wound cleaning and administration of PEP, to prevent rabies-related deaths. The two cases with unknown outcomes also point to potential gaps in follow-up and reporting systems, which need to be strengthened to ensure accurate monitoring and evaluation of rabies cases [10].

The Lefeisa rabies outbreak exposed critical deficiencies in veterinary care and diagnostic capabilities within the Somali region. While two cases were confirmed via rapid tests, numerous suspected cases likely went undiagnosed due to technical and facility limitations at local level. The community's proactive approach to seeking medical care after bites was commendable, yet their fear-driven, preemptive killing of suspected animals, though understandable, raises ethical concerns. This highlights the urgent need for community education on humane animal handling and proper rabies reporting protocols, fostering informed decision-making during crises.

The outbreak revealed significant gaps in veterinary and public health infrastructure. Limited diagnostic services heightened community fears and delayed essential public health responses. Furthermore, the scarcity of accessible post-exposure prophylaxis worsened the situation. Although Jigjiga Referral Hospital was relatively nearby at approximately 32 km and usually took about one hour to reach under normal conditions, poor road infrastructure, frequent traffic congestion, and routine security checkpoints often extended travel time significantly. These delays frequently resulted in the loss of critical time needed to provide timely treatment for bite victims.

Strengthening veterinary services and ensuring timely medical access are paramount for future initiatives. Building local healthcare capacity empowers communities to manage similar threats effectively, reducing reliance on actions that compromise animal welfare.

The low number of confirmed rabies cases underscores the need for enhanced animal health surveillance and data collection. In resource-limited settings, animal disease surveillance is often neglected, leading to preventable outbreaks. Robust surveillance systems, including sample collection and accurate reporting, are crucial for understanding rabies transmission dynamics. Integrating veterinary and public health efforts through a One Health approach facilitates interdisciplinary collaboration and timely interventions. This involves establishing community-based surveillance systems that engage pastoralists, animal health workers, and local clinics to report suspected cases in both domestic animals and wildlife. Joint training programs for medical and veterinary personnel can improve skills in rabies detection, case management, and community education, fostering stronger collaboration across sectors.

Creating a regional rabies task force composed of public health, animal health, and wildlife authorities will facilitate coordinated vaccination efforts and outbreak responses. Collaborating with environmental agencies to monitor wildlife behavior and ecological factors can provide critical insights into transmission dynamics. The unusual behavior of wild animals suggests ecological disruptions, emphasizing the need for understanding wildlife behavior and human-wildlife interactions to prevent zoonotic spillover. Raising awareness of wildlife vaccination and human-wildlife conflict risks can further mitigate rabies spread.

The Lefeisa outbreak necessitates comprehensive public health strategies focused on prevention, education, and ethical considerations. Future initiatives must prioritize zoonotic disease control while building trust with local communities through participatory decision-making.

## 5 Conclusion

The rabies outbreak in Lefeisa highlighted critical vulnerabilities across public health and veterinary systems, emphasizing the urgent need for targeted, localized interventions. The disproportionate impact on children, the predominance of dog bites, and the challenges in accessing timely post-exposure prophylaxis underscore the need for enhanced surveillance, robust dog vaccination campaigns, and community education. The identification of rabies in dogs and donkeys, alongside numerous suspected cases in various animal species, underscores the zoonotic risk. The community's proactive healthcare-seeking behavior after bite incidents was impressive, However, the preemptive killing of suspected animals due to fear raises ethical concerns, necessitating community education on humane animal handling and proper suspected rabies reporting for informed decision-making in crisis situations. To mitigate future rabies risks in Lefeisa and the broader Somali region, a comprehensive One Health approach is essential, including sustained dog vaccination campaigns, robust public awareness initiatives, improved access to post-exposure prophylaxis, and enhanced veterinary and human health surveillance systems. Addressing these deficiencies is critical for safeguarding both human and animal health and preventing future outbreaks in this vulnerable community.

## Ethical disclaimer

We are committed to promoting compassion and respect for all living beings. We recognize the ethical responsibility to treat animals humanely, especially in situations involving fear or crisis. We advocate for responsible decision-making that prioritizes the well-being of animals while ensuring community safety. As such, we emphasize the importance of education regarding humane animal handling practices and effective reporting of health concerns. Our goal is to foster a community that values empathy, informed action, and ethical considerations in every decision affecting animals and public health.

## CRediT authorship contribution statement

**Hassan Abdi Hussein:** Writing – review & editing, Writing – original draft, Supervision, Methodology, Investigation, Formal analysis, Data curation, Conceptualization. **Abdilahi Adan Ahad:** Writing – review & editing, Methodology, Investigation, Formal analysis. **Mohamed Hassen Abdilahi:** Investigation, Data curation.

## Funding

The authors (s) received no specific funding for this study. However, logistics were provided by Pastoral Development Bureau of Somali regional State.

## Declaration of competing interest

The author declares that there is no conflict of interest.

## Data Availability

Data will be made available on request.
